# Development and validation of a sentence-in-noise test for signal-to-noise ratio loss in adult normal-hearing and hearing-impaired listeners in South Africa

**DOI:** 10.4102/sajcd.v73i1.1193

**Published:** 2026-09-29

**Authors:** Alida Naudé, René Hornby, Rozelle Roets

**Affiliations:** 1 Division of Speech-Language and Hearing Therapy, Department of Health and Rehabilitation Sciences, Stellenbosch University, Tygerberg Campus Cape Town, South Africa; 2 Rene Hornby Oudioloë Inc., Pretoria, South Africa

**Keywords:** sentence-in-noise, speech-in-noise, audiology, Afrikaans, South African English, sentence recognition in babble noise, 4-talker babble noise, SNR loss

## Abstract

**Background:**

Difficulties understanding speech in noise are common in adults with hearing impairment. Yet, validated sentence-based speech-in-noise measures remain limited in South African clinical practice.

**Objectives:**

To validate two language-specific South African sentence-in-noise tests, the South African Sentence-in-Noise (SASIN) test and *Suid-Afrikaanse Sin-in-Geraas* (SASIG) test, by examining inter-list comparability and determining the signal-to-noise ratio (SNR) required for 50% correct sentence recognition (SNR-50).

**Method:**

A quantitative, retrospective validation study used routinely collected clinical data from adult first-language speakers of South African English (SASIN; *n* = 36) or Afrikaans (SASIG; *n* = 51) across multiple audiology sites. Participants completed five sentence lists in four-talker babble using fixed 5 dB SNR steps. Keyword scores were analysed for inter-list comparability and for deriving SNR-50 and SNR-loss values.

**Results:**

Friedman analyses indicated significant overall list effects for both tests. Bonferroni-adjusted pairwise comparisons indicated that only selected comparisons, primarily involving List 1, reached statistical significance, whereas most pairwise comparisons were not statistically significant. Overall, the findings supported broad inter-list comparability.

**Conclusion:**

The SASIN and SASIG tests demonstrated acceptable psychometric properties as language-specific sentence-in-noise measures for South African English- and Afrikaans-speaking adults, supporting their clinical use.

**Contribution:**

This study provides validated first-language sentence-in-noise tools and test-specific SNR-50 and SNR-loss metrics for adult audiological assessment in South Africa.

## Introduction

Pure-tone audiometry remains a foundational component of audiological assessment; however, hearing thresholds alone do not adequately reflect an individual’s ability to understand speech in everyday listening environments, particularly under acoustically adverse conditions (Humes et al., 2012; Plomp, 1978). Difficulties with speech understanding in noise (commonly referred to as speech-in-noise perception) are among the most frequently reported communication complaints in adults, affecting individuals with normal or near-normal audiograms as well as those with measurable hearing loss (Dillon et al., 2012; Kramer et al., 1995; Wilson & McArdle, 2005). As a result, contemporary audiological practice increasingly emphasises functional assessment approaches that extend beyond audibility to include speech perception under degraded listening conditions. The South African Sentence-in-Noise (SASIN) test and the *Suid-Afrikaanse Sin-in-Geraas* (SASIG) are designed to assess functional speech understanding in noise as a clinical outcome measure; in the present study, performance was interpreted in relation to peripheral hearing status and should not be interpreted as a direct measure of central auditory processing.

Within this functional framework, signal-to-noise ratio (SNR) testing has become a core component of adult speech audiometry. Rather than relying solely on speech recognition scores obtained in quiet, SNR-based measures quantify the listening conditions under which an individual can achieve a defined level of speech understanding, most commonly expressed as the SNR required for 50% correct recognition (SNR-50). International clinical guidelines and practice recommendations recognise SNR testing as an essential element of the basic audiological test battery, as it provides clinically meaningful information about real-world listening performance that cannot be inferred from pure-tone thresholds alone (British Society of Audiology, 2019; Dillon et al., 2012; Gatehouse & Noble, 2004).

The terms speech-in-noise and sentence-in-noise are used descriptively throughout this article to refer to functional speech perception under noisy conditions and do not denote a proprietary test or branded protocol.

Sentence-based SNR tests are regarded as particularly valuable because they more closely approximate everyday communication demands than isolated word recognition measures. Sentence perception in noise requires the integration of auditory processing, linguistic knowledge, working memory and attentional resources, especially when the competing signal consists of meaningful speech rather than steady-state noise (Pichora-Fuller et al., 2016; Plomp, 1978). Performance on sentence-in-noise tasks has been shown to correlate more strongly with self-reported communication difficulties, hearing-aid benefit and functional listening outcomes than speech recognition testing conducted in quiet (Humes, 2002; Kramer et al., 1995).

Internationally, several sentence-based SNR tests have been developed and validated for clinical use, including the Hearing in Noise Test (HINT), the Bamford–Kowal–Bench Speech-in-Noise Test (BKB-SIN) and the Quick Speech-in-Noise Test (QuickSIN) (Bench et al., 1979; Killion et al., 2004; Nilsson et al., 1994). These instruments are widely used to quantify SNR loss, support hearing-aid selection and verification and facilitate counselling regarding communication difficulties in noise. However, the validity of such measures is intrinsically linked to the linguistic, phonetic and prosodic properties of the test material. Speech perception in noise is language dependent, and the use of test materials developed for other linguistic contexts may compromise both the accuracy and interpretability of assessment outcomes (Gatehouse & Noble, 2004; Plomp, 1978; Smits et al., 2013).

In the South African context, these challenges are amplified by the country’s multilingual population and the limited availability of validated speech audiometry materials in local languages. There is a documented shortage of standardised speech audiometry materials and procedures across South Africa’s official languages, with the gap being particularly pronounced for sentence materials (Theunissen et al., 2011). Thesis-based work further suggests that sentence materials are rarely used in routine South African practice, with clinicians relying predominantly on spondee and monosyllabic word measures for diagnostic purposes (Scourfield, 2011). Where sentence measures have been reported, they have included instruments not developed for the South African population, and clinicians reported limited access to, or knowledge of, standardised local sentence tests (Roets, 2005). In addition, the routine use of speech-in-noise procedures in South Africa remains poorly characterised in the published survey literature, and informal approaches (e.g. applying speech materials standardised in quiet with background noise added) risk undermining standardisation assumptions and interpretability (Roets, 2005). This practice persists despite longstanding evidence that assessment in a patient’s first or most proficient language is essential for obtaining valid speech recognition results, particularly under adverse listening conditions (Carhart, 1965; Gatehouse & Noble, 2004).

South African English and Afrikaans exhibit distinct phonetic, lexical and prosodic characteristics that differ from the language varieties on which most international SNR tests are based. The use of imported sentence materials may therefore introduce unintended linguistic and dialectal biases, potentially resulting in poorer test performance that reflects language mismatch rather than auditory limitation. Such effects are especially problematic when SNR measures are used to inform clinical decision-making, counselling and amplification recommendations.

To address this gap, the SASIN test and the SASIG test were developed as linguistically and culturally appropriate sentence-based SNR assessment tools for adult patients in South Africa. Both tests were designed to quantify speech perception in the presence of four-talker babble noise, an informational masker that more closely reflects the acoustic and cognitive demands of everyday social listening environments than steady-state noise (Plomp, 1978; Simpson & Cooke, 2005). Although SASIN and SASIG share a common conceptual framework, they are distinct, language-specific instruments.

The following section describes the development of the SASIN and SASIG, including the linguistic design of the sentence materials, recording procedures, competing signal characteristics and the derivation of SNR-50 and SNR-loss metrics. Together, these elements provide the methodological foundation for subsequent evaluation of inter-list comparability, normative performance and clinical applicability.

The aims of this study were to (1) describe sentence-in-noise performance across varying SNRs for two language-specific sentence-in-noise tests, the SASIN test and the SASIG test, in adults with different hearing statuses; (2) estimate the SNR-50 and derive SNR-loss values relative to normal-hearing reference performance; and (3) evaluate inter-list comparability to determine whether the five sentence lists perform similarly under standardised clinical conditions.

## Development of the South African Sentence-in-Noise and *Suid-Afrikaanse Sin-in-Geraas*

### Conceptual framework

The SASIN and SASIG tests were developed using established principles of sentence-based SNR assessment, adapted for application within the South African linguistic context. Development focused on operationalising core design features known to support reliable, clinically efficient measurement of functional speech perception in noise.

The test framework was informed by widely used sentence-in-noise methodologies, including fixed SNR step presentation, keyword-based scoring and estimation of the SNR-50 (Killion et al., 2004; Nilsson et al., 1994; Tillman & Olsen, 1973). These principles were applied consistently across both language versions to ensure methodological comparability, while all linguistic materials and recordings were developed independently for SASIN and SASIG.

Both tests were designed for efficient clinical administration within routine adult audiological assessment. Fixed SNR steps were selected to allow rapid estimation of SNR-50 without adaptive tracking procedures. The use of sentence materials, rather than isolated words, was intended to capture functional speech understanding under acoustically adverse conditions.

Although the SASIN and SASIG share a common test architecture, they are distinct instruments. Each employs language-specific sentence material recorded by first-language speakers and is standardised using separate normative datasets. As such, performance metrics, including SNR-50 and derived SNR-loss values, are test specific and should be interpreted within the context of each instrument rather than compared directly across tests.

### Sentence construction and linguistic design

Sentence materials for the SASIN and SASIG were constructed to minimise the influence of non-auditory factors on performance while preserving ecological validity. Design decisions focused on controlling sentence length, syntactic complexity, semantic predictability and phonetic composition, all of which are known to influence speech-in-noise outcomes independently of auditory sensitivity.

Sentences were limited to 6–10 words to reduce working memory and executive processing demands, thereby ensuring that performance primarily reflected auditory–linguistic processing under degraded listening conditions. Excessively long or syntactically complex sentence structures were avoided, as increased cognitive load has been shown to disproportionately affect speech recognition in noise, particularly in listeners with hearing impairment or age-related cognitive changes (Akeroyd, 2008; Wingfield & Tun, 2007).

All sentences were grammatically correct and representative of everyday spoken language while maintaining low contextual predictability. Keywords could not be reliably inferred from surrounding linguistic context, reducing semantic cueing and limiting ceiling effects at favourable SNRs. Nonsensical sentence constructions were avoided to maintain participant engagement and functional relevance.

Each sentence contained five predefined keywords, which served as the scoring units. Keyword-based scoring provides sensitivity to performance changes across SNRs while remaining feasible for routine clinical administration and has been widely adopted in sentence-in-noise test design (Killion et al., 2004; Nilsson et al., 1994).

Language-specific phonetic balance was addressed independently for each test. For the SASIG, keyword selection was informed by established principles of phonetic representativeness in Afrikaans speech audiometry, drawing on the *Foneties Verteenwoordigende Eenlettergrepige Woordelys in Afrikaans* (FVEWA) (Naudé, 2018). For the SASIN, isophonemic principles were applied to ensure balanced phoneme representation appropriate to South African English, informed by the established iso-phonemic English Arthur Boothroyd wordlists (Boothroyd, 1968, 1985).

All sentence materials were reviewed by a qualified linguist to confirm grammatical accuracy, phonetic balance and naturalness of production. Minor revisions were implemented where necessary prior to recording, without altering sentence length or contextual predictability.

### Test lists

Five test lists were developed for each instrument. Each list consisted of six sentences, yielding a total of 30 scored keywords per list. This structure was selected to balance test efficiency with sufficient sampling of speech material to support inter-list comparability and reliable estimation of SNR-50 values, consistent with established sentence-in-noise test designs (Nilsson et al., 1994; Smits et al., 2013).

### Recording procedures

Sentence recordings were produced by male first-language speakers of South African English for the SASIN and Afrikaans for the SASIG. The use of a single male speaker for each test was intended to minimise inter-speaker variability and to provide a stable acoustic reference for clinical assessment, as variability in speaker characteristics has been shown to influence speech recognition performance in noise (Bent & Bradlow, 2003; Bradlow et al., 1996).

All recordings were conducted in a professional sound studio by an experienced sound engineer using calibrated recording equipment. Speech signals were edited, levelled and normalised to ensure consistent intensity across sentences and lists. Final audio files were reviewed and approved by the test developers prior to integration with the competing signal.

### Competing signal: Four-talker babble noise

Both the SASIN and SASIG employ four-talker babble noise as the competing signal. The babble noise was created using recordings from four adult speakers, comprising two male and two female talkers, each reading unrelated passages aloud in their respective first language. The individual recordings were mixed to produce a continuous, unintelligible speech background.

Four-talker babble noise functions primarily as an informational masker, introducing linguistic and cognitive interference in addition to energetic masking (Brungart et al., 2001; Simpson & Cooke, 2005). Informational masking has been shown to place increased demands on central auditory and cognitive processing and to more closely approximate everyday listening environments such as group conversations (Kidd et al., 2002; Pichora-Fuller et al., 2016). The use of four-talker babble noise was therefore intended to enhance the ecological validity and clinical relevance of the SASIN and SASIG compared to steady-state noise maskers.

### Signal-to-noise ratio presentation

Sentences were presented at a fixed speech level, while the level of the four-talker babble noise was varied to create different SNRs. The SNR range extended from +25 dB to 0 dB, encompassing listening conditions from relatively favourable to highly challenging. Signal-to-noise ratios were adjusted in fixed 5 dB steps, and participant performance was quantified based on the total number of keywords correctly repeated across the six sentences in each list. This stepwise presentation approach enables efficient estimation of SNR-50 values without the need for adaptive tracking procedures and is consistent with established clinical sentence-in-noise methodologies (Killion et al., 2004; Smits et al., 2013).

### Estimation of signal-to-noise ratio-50

The SNR-50 was estimated using principles derived from the method described by Tillman and Olsen (1973) for speech reception threshold estimation. This approach has been widely adopted in sentence-in-noise testing because it permits reliable threshold estimation using a limited number of test items and a simple arithmetic calculation.

For the SASIN and SASIG, the starting SNR was set at +25 dB. A correction factor of +2.5 dB, corresponding to half the SNR step size, was applied to account for the discrete nature of the stepwise procedure. The SNR-50 was calculated as: SNR-50 = 25 + 2.5 − (total number of keywords correctly repeated), which simplifies to: SNR-50 = 27.5 − (total correct).

### Derivation of signal-to-noise ratio loss

To facilitate clinical interpretation, SNR loss was derived by referencing individual SNR-50 values to the empirically determined normal-hearing performance for each test. During standardisation, the mean and median SNR-50 for normal-hearing participants were −1 dB, indicating that the SASIN and SASIG were less difficult for normal-hearing participants than some established sentence-in-noise tests.

Signal-to-noise ratio loss was calculated as the difference between the individual SNR-50 and the normal-hearing reference value, such that: SNR loss = SNR-50 − (−1). Substituting the SNR-50 estimation yields: SNR loss = (27.5 − total correct) + 1, which simplifies to:

SNR loss = 28.5 − (total number of keywords correctly repeated).

This approach ensures that SNR-loss values accurately reflect deviation from test-specific normal-hearing performance, rather than applying correction factors derived from other sentence-in-noise tests with different difficulty characteristics.

### Clinical categorisation of signal-to-noise ratio loss

Signal-to-noise ratio-loss values provide a clinically interpretable index of functional listening difficulty by quantifying the additional SNR required, relative to normal-hearing participants, to achieve 50% keyword recognition. Although categorical descriptors of SNR loss (e.g. mild–severe difficulty) are commonly used in clinical practice, establishing test-specific severity cut-offs for the SASIN and SASIG requires larger normative samples and outcome-linked validation. Accordingly, the present study reports continuous SNR-loss values, and future work should focus on validating clinically meaningful severity categories for these instruments.

## Research methods and design

### Study design

A quantitative, retrospective validation study was conducted using routinely collected clinical data to examine the psychometric performance of two sentence-based SNR tests: the SASIN test and the SASIG test. The validation objectives were to examine inter-list comparability within each test and to confirm the SNR required for 50% correct keyword recognition (SNR-50), supporting calculation of test-specific SNR-loss outcomes as described in the Development section.

### Data sources and test administration sites

Data were collected across 11 A. van der Merwe Group practices and ear institutes located in six South African provinces (North West, KwaZulu-Natal, Western Cape, Gauteng, Eastern Cape and Limpopo) as part of routine adult audiological assessment. All sites followed a standardised protocol for administering and scoring the SASIN and SASIG tests. Testing was conducted in sound-treated rooms using calibrated one- or two-channel diagnostic audiometers, with recorded test material presented via an external playback source.

### Participants

Clinical records were included for adults aged 18 years and older who completed the SASIN or SASIG. Inclusion criteria were normal hearing sensitivity or bilateral symmetrical sensorineural hearing loss. Hearing status was classified into three subgroups for analysis: normal hearing, mild sensorineural hearing loss and moderate-to-severe sensorineural hearing loss, based on routine pure-tone audiometry.

Exclusion criteria, applied per the test protocol, were abnormal otoscopic findings; tympanometry other than Type A (i.e. Type B or Type C), conductive or mixed hearing loss, single-sided deafness and asymmetrical hearing loss. Only participants with normal middle-ear function (Type A) were included. Asymmetry was defined as an interaural threshold difference of ≥ 30 dB HL at three consecutive frequencies. Separate datasets were analysed for each language test (SASIN: *n* = 36; SASIG: *n* = 51).

### Test procedure

All participants completed the full protocol of five sentence lists for the relevant test. Stimuli were presented binaurally via headphones or insert earphones. Sentence presentation level was fixed throughout testing, while the level of the competing four-talker babble noise varied across sentences to create the required SNR conditions.

For participants with pure-tone averages (PTA; 500 Hz, 1000 Hz and 2000 Hz) ≤ 45 dB HL, sentences were presented at 70 dB HL; if this was judged uncomfortably loud, presentation level was reduced to 60 dB HL. For participants with PTA ≥ 50 dB HL, presentation level was adjusted to a participant-judged ‘loud but comfortable’ level to ensure audibility without discomfort. Participants listened to standardised recorded instructions and completed two practice sentences prior to formal testing. They were instructed to repeat each sentence verbatim.

### Test structure and scoring

Each test comprised five lists of six sentences. Five predetermined keywords per sentence were scored, yielding a maximum of 30 keywords per list. Each sentence was presented at a different fixed SNR, progressing in 5 dB steps from +25 dB SNR for the first sentence to 0 dB SNR for the final sentence. Consequently, each 5 dB SNR level contained one sentence with five scored keywords, resulting in a total of 30 scored keywords distributed across the six SNR conditions within each list. Sentences were presented in four-talker babble noise with fixed 5 dB SNR steps spanning +25 dB SNR – 0 dB SNR. Responses were scored live by the administering audiologist, with one point awarded per correctly repeated keyword. The primary outcome variable was the total number of correctly repeated keywords per list.

Signal-to-noise ratio-50 and SNR-loss outcomes were derived using the test-specific procedures described in the Development section. Percentage-correct scores for the normal-hearing subgroup were plotted against SNR, and the SNR corresponding to 50% correct keyword recognition (SNR-50) was estimated by linear interpolation of the psychometric function. This estimated normal-hearing reference value (−1 dB for both the SASIN and SASIG) was subsequently used to calculate SNR loss according to the established test-specific derivation: SNR loss = 28.5 − (total number of keywords correctly repeated).

### Statistical analysis

Analyses were conducted separately for the SASIN and SASIG datasets. Descriptive statistics (mean, standard deviation) were calculated for keyword totals for each list and for each hearing-status subgroup. Inter-list comparability was assessed using Friedman tests, the nonparametric analogue of repeated-measures analyses of variance (ANOVA), given the ordinal nature of keyword scores and violations of normality assumptions. Where the Friedman test indicated a significant overall effect, post hoc pairwise comparisons were conducted using Bonferroni-adjusted significance levels. Descriptive statistics (means and standard deviations) were calculated for each list and hearing-status subgroup. Statistical significance was set at *p* < 0.05 after adjustment for multiple comparisons.

### Ethical considerations

Data were obtained from routine clinical assessments. Ethical approval for the study was granted by the Stellenbosch University Health Research Ethics Committee (Reference No: N26/04/040). A waiver of informed consent was approved because of the retrospective nature of the study and the use of de-identified (link-anonymised) clinical data. No identifiable information was included in the dataset, and all data were managed in accordance with ethical principles of confidentiality, data protection and the *Protection of Personal Information Act (POPIA), Act No. 4 of 2013*. The study involved no deviation from standard clinical care and posed no additional risk to participants.

## Results

Data from adult participants who completed either the SASIN test or the SASIG test as part of routine clinical assessment were included in the analysis. Separate datasets were analysed for each test. Participants were categorised into three hearing-status groups, namely normal hearing, mild hearing loss and moderate–severe hearing loss, based on pure-tone audiometric findings. All participants met the predefined inclusion and exclusion criteria, including normal otoscopic findings and Type A tympanograms, and all participants completed all five sentence lists for the relevant test.

### Sentence-in-noise performance across signal-to-noise ratios

Mean sentence-in-noise performance, expressed as percentage of correctly identified keywords, is shown as a function of SNR for the SASIG in Figure 1 and for the SASIN in Figure 2, with descriptive values summarised in Table 1 and Table 2. For both tests, sentence recognition performance decreased systematically as SNR became less favourable across all hearing-status groups. Normal-hearing participants demonstrated the highest keyword recognition scores at each SNR, followed by participants with mild hearing loss, while participants with moderate–severe hearing loss showed the lowest performance, particularly at lower SNRs. As hearing loss severity increased, performance curves were progressively shifted towards more favourable SNRs, indicating a greater SNR requirement to achieve comparable levels of sentence recognition. This pattern was consistent across both language tests and across all sentence lists.

**FIGURE 1:**
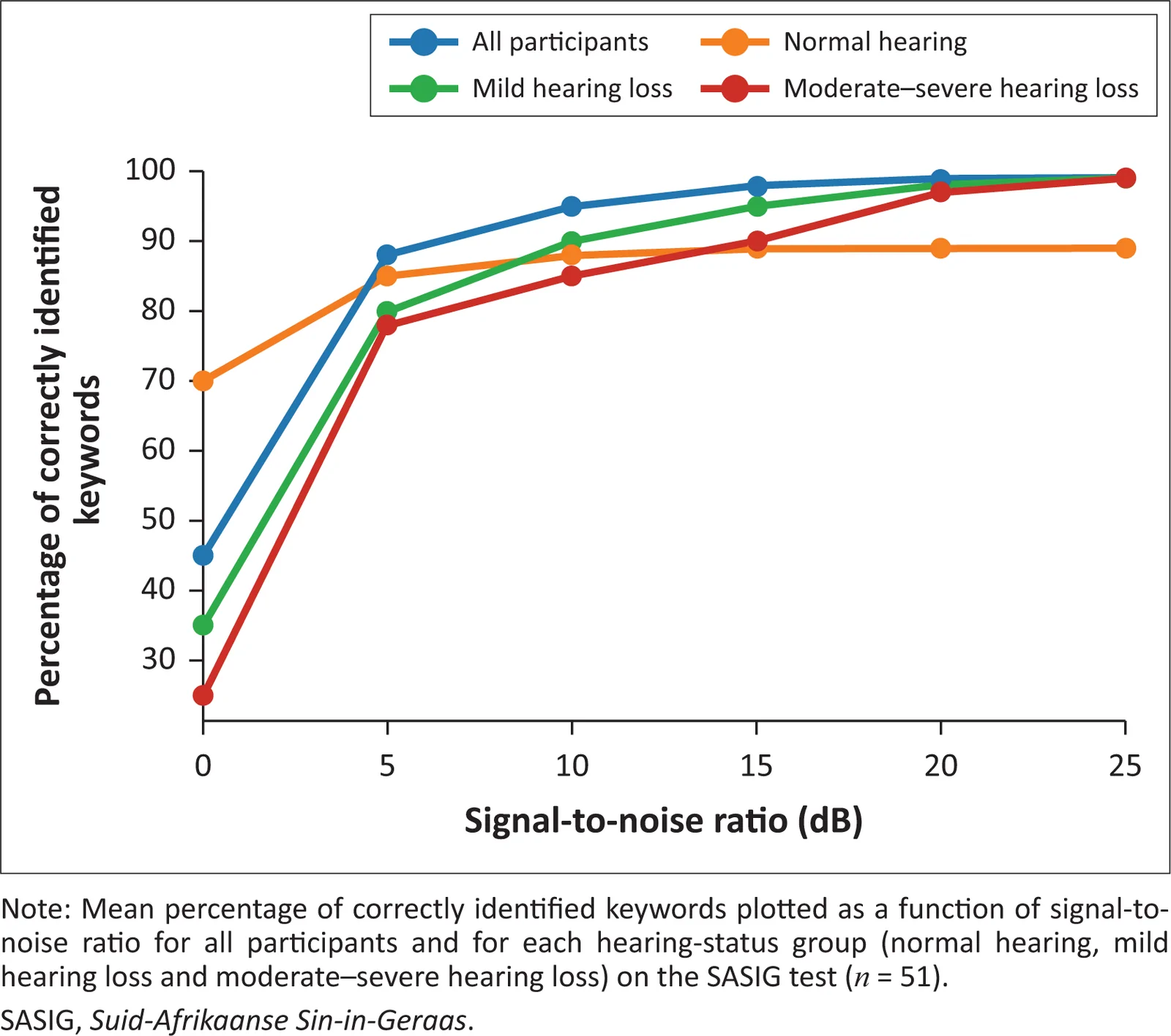
Sentence-in-noise performance for the *Suid-Afrikaanse Sin-in-Geraas* test across signal-to-noise ratios for all participants and by hearing-status group.

**FIGURE 2:**
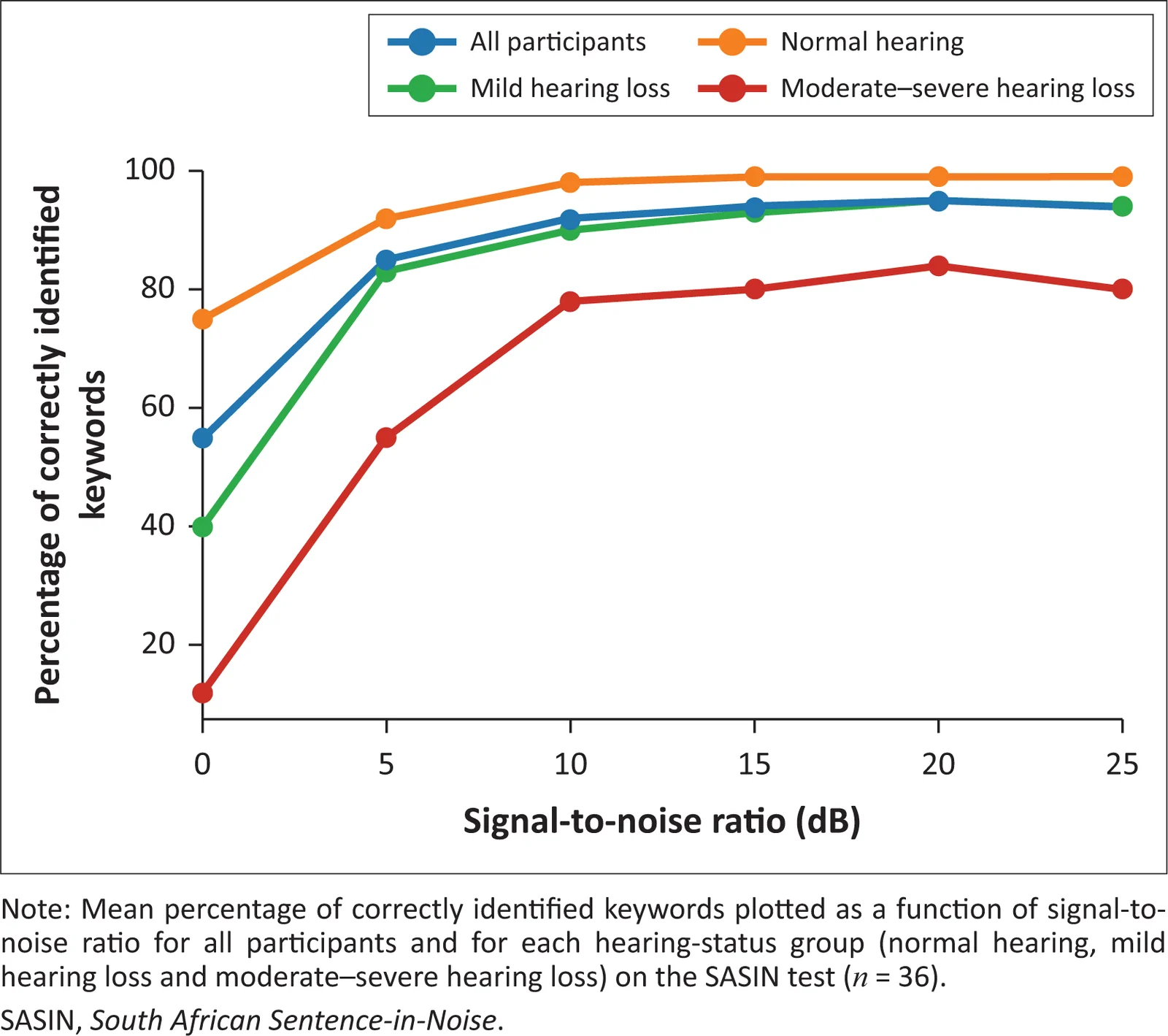
Sentence-in-noise performance for the South African Sentence-in-Noise test across signal-to-noise ratios for all participants and by hearing-status group.

**TABLE 1:** Mean percentage of correctly identified keywords for each sentence presented at successive signal-to-noise ratios in the *Suid-Afrikaanse Sin-in-Geraas*.

SNR (dB)	All participants	Normal hearing	Mild hearing loss	Moderate–severe hearing loss
0	45	70	35	25
5	88	85	80	78
10	95	88	90	85
15	98	89	95	90
20	99	89	98	97
25	99	89	99	99

Note: Values represent mean percentage of correctly identified keywords at each signal-to-noise ratio condition. Each SNR condition comprised one sentence containing five scored keywords, and each complete test list consisted of six sentences (30 scored keywords) presented sequentially across six fixed SNR levels (+25 dB, +20 dB, +15 dB, +10 dB, +5 dB and 0 dB SNR). Data are shown for all participants combined and stratified by hearing-status group (normal hearing, mild hearing loss and moderate–severe hearing loss).

SNR, signal-to-noise ratio.

**TABLE 2:** Mean percentage of correctly identified keywords for each sentence presented at successive signal-to-noise ratios in the South African Sentence-in-Noise.

SNR (dB)	All participants	Normal hearing	Mild hearing loss	Moderate–severe hearing loss
0	55	75	40	12
5	85	92	83	55
10	92	98	90	78
15	94	99	93	80
20	95	99	95	84
25	94	99	94	80

Note: Values represent mean percentage of correctly identified keywords at each signal-to-noise ratio condition. Each SNR condition comprised one sentence containing five scored keywords, and each complete test list consisted of six sentences (30 scored keywords) presented sequentially across six fixed SNR levels (+25 dB, +20 dB, +15 dB, +10 dB, +5 dB and 0 dB SNR). Data are shown for all participants combined and stratified by hearing-status group (normal hearing, mild hearing loss and moderate–severe hearing loss).

SNR, signal-to-noise ratio.

### Estimation of signal-to-noise ratio-50 and signal-to-noise ratio loss

Signal-to-noise ratio-50 values were estimated using the predefined fixed-step calculation method. Normal-hearing reference performance for both SASIG and SASIN was −1 dB SNR, as established in the respective test standardisation datasets.

Estimated SNR-50 values increased systematically with degree of hearing loss for both tests. Normal-hearing participants achieved the 50% criterion at the most adverse SNRs, while participants with mild hearing loss required more favourable SNRs. Participants with moderate–severe hearing loss demonstrated the greatest SNR requirements to achieve equivalent performance. Derived SNR-loss values for each hearing-status group are presented in Table 3.

**TABLE 3:** Estimated signal-to-noise ratio-50 and derived signal-to-noise ratio loss for the *Suid-Afrikaanse Sin-in-Geraas* and South African Sentence-in-Noise by hearing-status group.

Test	Group	Mean SNR-50 (dB)	Mean SNR loss (dB)
SASIG	Normal hearing	−1.0	0.0
SASIG	Mild hearing loss	+0.5	+1.5
SASIG	Moderate–severe loss	+2.0	+3.0
SASIN	Normal hearing	−1.0	0.0
SASIN	Mild hearing loss	+1.0	+2.0
SASIN	Moderate–severe loss	+3.5	+4.5

Note: SNR-50 represents the estimated signal-to-noise ratio at which 50% of keywords were correctly identified. The normal-hearing reference SNR-50 was estimated from the psychometric performance function by linear interpolation and was subsequently used to derive SNR-loss values. SNR loss was calculated relative to the normal-hearing reference value of −1 dB SNR for each test. Positive SNR-loss values indicate the additional signal-to-noise ratio required relative to normal-hearing listeners to achieve 50% sentence recognition, with higher values reflecting greater difficulty understanding speech in noise.

SASIG, *Suid-Afrikaanse Sin-in-Geraas*; SASIN, South African Sentence-in-Noise; SNR, signal-to-noise ratio.

### Inter-list comparability

Inter-list comparability was examined using Friedman tests conducted separately for the SASIN and SASIG. For the SASIG, a statistically significant overall effect of list order was observed (*χ*^2^[4] = 26.49, *p* < 0.001). Bonferroni-adjusted post hoc pairwise comparisons indicated that only selected comparisons, primarily involving List 1, reached statistical significance after adjustment for multiple comparisons, whereas most pairwise comparisons were not statistically significant. For the SASIN, a similar pattern was found, with a significant overall list-order effect (*χ*^2^[4] = 18.97, *p* < 0.001). Bonferroni-adjusted post hoc pairwise comparisons similarly showed that only selected comparisons, primarily involving List 1, reached statistical significance after adjustment for multiple comparisons, whereas most pairwise comparisons were not statistically significant. Overall, these findings indicate broadly comparable performance across the five sentence lists for both the SASIG and SASIN, although small differences involving List 1 were identified. Mean list performance and rank-order patterns are illustrated in Figure 3.

**FIGURE 3:**
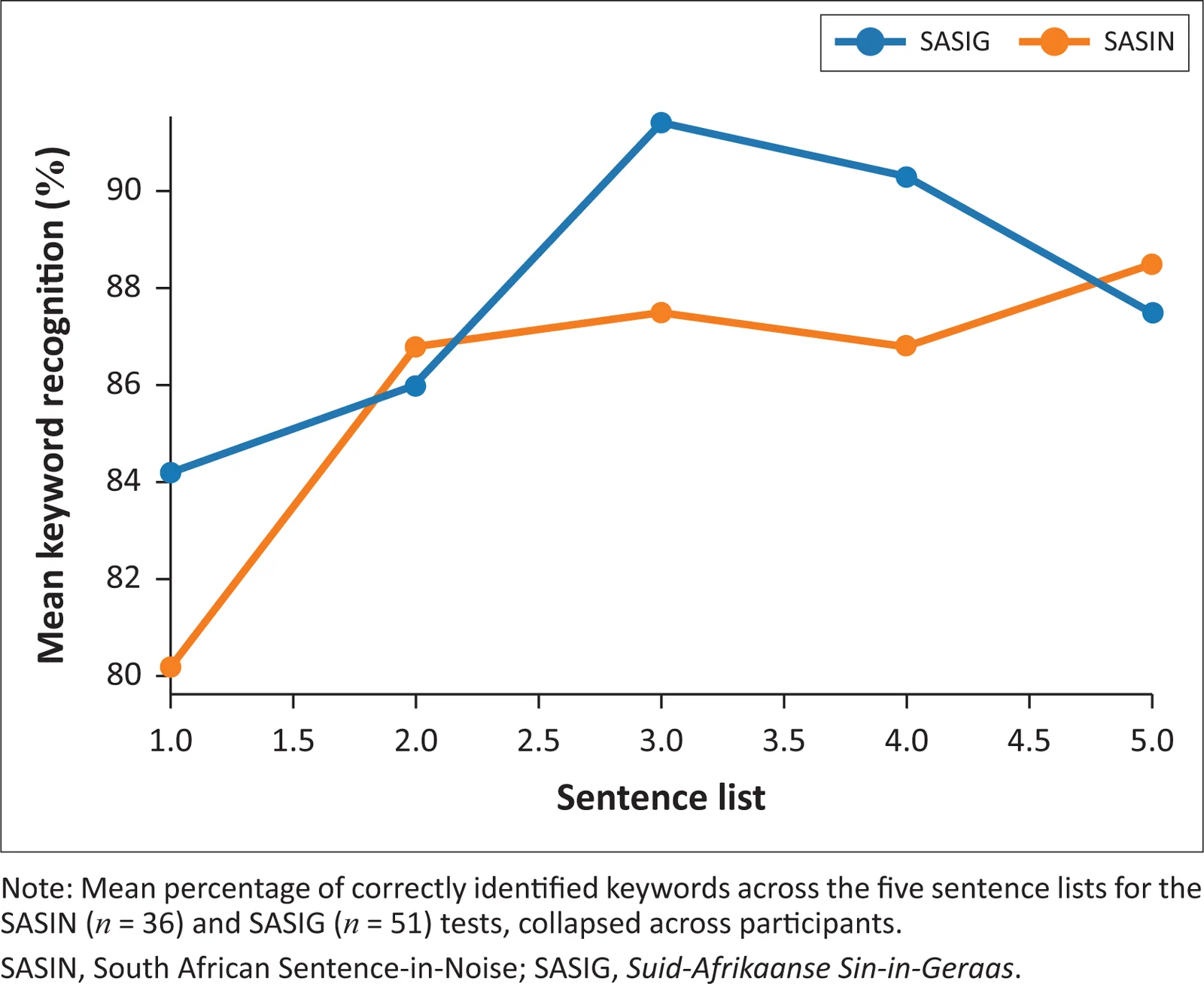
Mean keyword recognition across the five sentence lists for the *Suid-Afrikaanse Sin-in-Geraas* and South African Sentence-in-Noise, demonstrating inter-list comparability.

## Discussion

This study evaluated the psychometric properties of two language-specific sentence-in-noise tests developed for adult audiological assessment in South Africa, namely the SASIN and SASIG. In line with the study aims, sentence-in-noise performance was examined across SNRs, test-specific SNR-50 and SNR-loss values were derived, and inter-list comparability was evaluated to determine whether the sentence lists demonstrated comparable performance for routine clinical use. Overall, the findings support the known-groups validity, clinical applicability and practical utility of both instruments as measures of functional speech understanding in noise.

### Inter-list comparability

The five sentence lists within each test demonstrated comparable performance patterns, indicating that no list consistently produced substantially different scores under the standardised test protocol. Mean list performance and rank-order patterns were consistent across lists for both the SASIN and SASIG, with no evidence of clinically meaningful differences in list difficulty. This pattern was also observed across hearing-status subgroups, including normal-hearing listeners and those with mild and moderate–severe hearing loss, although subgroup findings should be interpreted cautiously because of smaller sample sizes in some groups.

Although the Friedman analyses identified statistically significant overall list effects, Bonferroni-adjusted post hoc comparisons demonstrated that only selected pairwise differences reached statistical significance, primarily involving List 1, while most other comparisons were not statistically significant. These findings indicate broadly comparable list performance but do not establish formal statistical equivalence.

The observed comparability in list performance has important practical implications. It suggests that clinicians may select different sentence lists for initial assessment and follow-up testing with minimal evidence of systematic list-related bias, supporting the practical use of different sentence lists during serial clinical assessments. Although small differences involving List 1 were identified, these are likely attributable to familiarisation effects rather than clinically meaningful differences in sentence difficulty.

### Sentence-in-noise performance and signal-to-noise ratio-50

Both tests demonstrated the expected monotonic decline in sentence recognition performance as listening conditions became more adverse. Performance curves showed a systematic horizontal shift with increasing hearing loss severity, reflecting greater SNR demands to achieve equivalent levels of sentence recognition. This pattern is consistent with established models of suprathreshold speech perception in noise, in which reduced audibility, suprathreshold processing and increased listening effort contribute to performance under adverse acoustic conditions.

The independently derived SNR-50 value of approximately −1 dB for both the SASIN and SASIG is consistent with the range reported for established sentence-in-noise tests in other languages, including the HINT and related sentence-based measures, which typically report SNR-50 values between approximately −2 dB and +2 dB depending on the language, masker type and test procedure (Killion et al., 2004; Kollmeier et al., 2015; Nilsson et al., 1994). This comparison is intended only to demonstrate that the estimated thresholds are methodologically and clinically plausible within the broader speech-in-noise literature. Because SNR-50 values are influenced by language-specific materials, masking noise, scoring procedures and psychometric characteristics, they should not be interpreted as evidence of equivalence or used for direct comparison across different speech-in-noise tests.

The estimated normal-hearing SNR-50 of approximately −1 dB was derived from the psychometric performance function using linear interpolation and therefore represents an estimated threshold rather than a directly tested presentation level. Because the lowest SNR administered during testing was 0 dB, the estimated SNR-50 lies marginally below the measured range. This interpolation provides a practical normal-hearing reference for calculating SNR loss but should be interpreted as an estimate rather than a directly observed threshold. Future studies incorporating additional negative SNR presentation levels would enable direct empirical verification of the estimated SNR-50.

An interesting observation was that normal-hearing participants demonstrated slightly lower percentage-correct scores on the SASIG than on the SASIN at equivalent SNRs, despite both instruments yielding an estimated normal-hearing reference SNR-50 of approximately −1 dB. This finding suggests that subtle language-specific differences in sentence structure, phonetic composition or lexical characteristics may influence the shape of the psychometric function without materially affecting the overall reference threshold. Because the present study was not designed to investigate language-related performance differences, this observation should be interpreted cautiously. Future studies using larger normative samples should examine whether this represents a consistent language-specific characteristic or reflects normal sampling variation.

The clear separation in performance between normal-hearing and hearing-impaired participants across SNR demonstrates that both the SASIN and SASIG were able to discriminate between groups expected to differ in speech-in-noise performance. While these findings support the known-groups validity and clinical applicability of the two tests, further studies incorporating additional criterion measures, such as self-reported communication outcomes or comparison with established speech-in-noise assessments, are required to establish formal construct validity.

### Clinical implications

The SASIN and SASIG provide clinicians with validated, first-language-specific tools for assessing functional speech understanding in noise within routine adult audiological practice. Sentence-in-noise testing captures communication difficulties that are often underestimated by pure-tone audiometry or speech recognition testing conducted in quiet, thereby offering clinically meaningful insight into everyday listening challenges.

The use of four-talker babble noise as the competing signal enhances ecological validity by approximating common social listening environments and introducing informational masking that more closely reflects everyday listening conditions. This allows clinicians to assess speech perception under conditions that more closely resemble real-world communication, supporting more meaningful interpretation of test outcomes.

The derivation of SNR-loss values provides a clinically interpretable metric that can be used to support patient counselling, facilitate explanation of listening difficulties in noisy environments and guide discussions regarding amplification strategies, directional microphone technology and assistive listening devices. By quantifying the additional SNR required for effective sentence recognition, clinicians are better equipped to set realistic expectations and align intervention recommendations with individual listening needs.

From a practical perspective, both tests are straightforward to administer, require minimal additional equipment beyond standard audiometric setups, and are well suited for integration into routine clinical workflows across diverse practice settings. The demonstrated inter-list comparability further supports flexible administration, with little evidence of clinically meaningful differences in list difficulty under the standardised protocol.

Importantly, assessing speech-in-noise performance using first-language sentence material supports more accurate interpretation of results by minimising linguistic confounds. This facilitates clearer differentiation between auditory-perceptual limitations and language-related influences on performance, contributing to more patient-centred clinical decision-making and more effective counselling regarding everyday communication challenges.

### Strengths and limitations

A key strength of this study is the validation of two language-specific sentence-in-noise instruments using real-world clinical data, enhancing the ecological validity of the findings. The study included participants with normal hearing sensitivity as well as mild and moderate-to-severe sensorineural hearing loss for both the SASIG (*n* = 51; normal hearing = 23, mild hearing loss = 21, moderate-to-severe hearing loss = 7) and the SASIN (*n* = 36; normal hearing = 19, mild hearing loss = 11 and moderate-to-severe hearing loss = 6). The inclusion of participants across a range of hearing sensitivities supports the clinical applicability of the findings.

Some limitations should be acknowledged. The retrospective design limited control over certain test-administration variables across sites. Although the overall sample sizes were appropriate for this initial validation study, the moderate-to-severe hearing loss subgroups were relatively small (SASIG: *n* = 7; SASIN: *n* = 6), which may limit the precision and generalisability of subgroup-specific findings. Cognitive factors known to influence speech-in-noise performance were not formally assessed and could not be controlled for in the analyses.

In addition, inclusion of highly favourable SNR conditions such as +25 dB SNR contributed limited incremental information for some participants; future refinements may benefit from inclusion of more challenging SNRs such as −5 dB SNR to enhance discrimination at lower performance levels. The present study evaluated binaural performance only, and ear-specific SNR-loss patterns were not examined.

### Recommendations for future research

Future studies employing prospective designs with larger and more balanced samples would strengthen normative data and subgroup analyses. Investigation of test–retest reliability, aided versus unaided performance and sensitivity to hearing-aid signal-processing features would further extend the clinical evidence base. Expansion of sentence-in-noise testing to additional South African languages, validation of the SASIN and SASIG in English second-language speakers and other multilingual populations and exploration of ear-specific SNR-loss measures represent important areas for future development.

## Conclusion

The SASIN and SASIG demonstrated acceptable psychometric properties as language-specific sentence-in-noise tests for use in South African audiology practice. The findings support the clinical use of the SASIN for adults whose first language is South African English and the SASIG for adults whose first language is Afrikaans. Both instruments demonstrated inter-list comparability and predictable performance patterns across SNRs, with an estimated normal-hearing reference SNR-50 of approximately −1 dB.

By enabling assessment of functional speech understanding in noise in a patient’s first language, the SASIN and SASIG address a longstanding gap in locally available speech audiometry tools. Their application has the potential to enhance diagnostic accuracy, guide evidence-based intervention and support patient-centred care in a multilingual clinical context. However, the performance and validity of these instruments in English second-language speakers and other multilingual populations have not yet been established and should be investigated in future research.
